# Healthy obesity and objective physical activity^1^,^2^,^3^

**DOI:** 10.3945/ajcn.115.110924

**Published:** 2015-07-08

**Authors:** Joshua A Bell, Mark Hamer, Vincent T van Hees, Archana Singh-Manoux, Mika Kivimäki, Séverine Sabia

**Affiliations:** 4 Department of Epidemiology & Public Health, University College London, London, United Kingdom;; 5 National Centre for Sport & Exercise Medicine, Loughborough University, Leicestershire, United Kingdom;; 6 MoveLab—Physical Activity and Exercise Research, Institute of Cellular Medicine, Newcastle University, Newcastle upon Tyne, United Kingdom;; 7 INSERM, Centre for Research in Epidemiology and Population Health, Villejuif, France; and; 8 University Versailles St-Quentin, Boulogne-Billancourt, France

**Keywords:** obesity, metabolic risk factor clustering, metabolic health, physical activity, epidemiology

## Abstract

**Background:** Disease risk is lower in metabolically healthy obese adults than in their unhealthy obese counterparts. Studies considering physical activity as a modifiable determinant of healthy obesity have relied on self-reported measures, which are prone to inaccuracies and do not capture all movements that contribute to health.

**Objective:** We aimed to examine differences in total and moderate-to-vigorous physical activity between healthy and unhealthy obese groups by using both self-report and wrist-worn accelerometer assessments.

**Design:** Cross-sectional analyses were based on 3457 adults aged 60–82 y (77% male) participating in the British Whitehall II cohort study in 2012–2013. Normal-weight, overweight, and obese adults were considered “healthy” if they had <2 of the following risk factors: low HDL cholesterol, hypertension, high blood glucose, high triacylglycerol, and insulin resistance. Differences across groups in total physical activity, based on questionnaire and wrist-worn triaxial accelerometer assessments (GENEActiv), were examined by using linear regression. The likelihood of meeting 2010 World Health Organization recommendations for moderate-to-vigorous activity (≥2.5 h/wk) was compared by using prevalence ratios.

**Results:** Of 3457 adults, 616 were obese [body mass index (in kg/m^2^) ≥30]; 161 (26%) of those were healthy obese. Obese adults were less physically active than were normal-weight adults, regardless of metabolic health status or method of physical activity assessment. Healthy obese adults had higher total physical activity than did unhealthy obese adults only when assessed by accelerometer (*P* = 0.002). Healthy obese adults were less likely to meet recommendations for moderate-to-vigorous physical activity than were healthy normal-weight adults based on accelerometer assessment (prevalence ratio: 0.59; 95% CI: 0.43, 0.79) but were not more likely to meet these recommendations than were unhealthy obese adults (prevalence ratio: 1.26; 95% CI: 0.89, 1.80).

**Conclusions:** Higher total physical activity in healthy than in unhealthy obese adults is evident only when measured objectively, which suggests that physical activity has a greater role in promoting health among obese populations than previously thought.

## INTRODUCTION

The economic burden of chronic disease attributable to obesity in developed countries is immense, with annual treatment costs projected to increase by US$66 billion in the United States and by £2 billion in the United Kingdom by 2030 (1). Partitioning the components of metabolic risk factor clustering from BMI allows for the stratification of chronic disease risk in epidemiologic research and for the prioritization of resources in clinical settings (2). Meta-analyses indicate that obese adults who are metabolically healthy have a risk of type 2 diabetes (3) and cardiovascular disease and mortality (4) that is intermediate between that of healthy normal-weight and unhealthy obese adults. This suggests that health risks for this group may not be eliminated, but sizable benefits may still be realized by promoting transitions from unhealthy obesity into its healthier counterpart.

Regular engagement in physical activity is known to protect against development of metabolic risk factor clustering, type 2 diabetes, and cardiovascular disease, with the degree of benefit proportional to the intensity of activity performed (5, 6). However, support for physical activity as a modifiable determinant of metabolic phenotypes of obesity has been inconsistent. Although studies to date have found no differences in total physical activity between healthy and unhealthy obese groups (7, 8), healthy obese men and women have reported higher moderate-to-vigorous physical activity than do their unhealthy obese counterparts in some studies (7, 9) but not in others (10, 11).

Comparisons of total and moderate-to-vigorous physical activity between metabolic phenotypes of obesity have relied on self-reported questionnaire-based measures of physical activity (7–11), which have only modest correlations with objective assessments (12, 13), possibly because of measurement errors related to inaccurate recall and social desirability (13). Previous studies reporting weak or null findings may have been unable to detect true differences in physical activity between obese groups due to reliance on imprecise measures of activity. Light-intensity physical activity, measured objectively, has been shown to reduce the risk of metabolic risk factor clustering independent of moderate-to-vigorous intensity activity (14). Current methods of distinguishing light intensity from sedentary time and moderate intensity activities are limited (15); however, light activities are captured in measures of total physical activity. Newly developed triaxial accelerometers have the potential to capture total physical activity in a more complete manner by recording incidental movements (16) and thus may offer valuable insight into the role of physical activity as a modifiable determinant of healthy obesity and of reductions in associated disease burden.

The current study aimed to determine whether adults with healthier metabolic phenotypes of obesity are more active than their unhealthy counterparts and whether this extends to a greater likelihood of meeting recommendations for moderate-to-vigorous physical activity by using novel wrist-worn triaxial accelerometer assessments of physical activity in addition to traditional questionnaire assessments in a large population-based study.

## METHODS

### Study population

The Whitehall II cohort study, based on government employees, recruited 10,308 men and women in 1985–1988 (17). Data from the 2012–2013 phase of assessment were used for the current analyses. Participants provided written informed consent. Ethical approval was obtained from the University College London research ethics committee.

### Questionnaire-assessed physical activity

Total physical activity was assessed by a modified version of the validated Minnesota Leisure Time Physical Activity Questionnaire (18, 19). Twenty items covered the amount of time spent in walking, in sports, in gardening, doing housework, in do-it-yourself activity, and in other activities through 2 open-ended questions. Participants were required to take into account activity patterns over the past 4 wk to indicate usual activity and provide the total number of hours spent in each activity per week. A metabolic equivalent (MET) value was assigned to each activity by using a compendium of activity energy costs (20). Total physical activity was estimated as MET-h/wk, the sum of the product of the intensity (MET) and weekly duration (h/wk) of all reported activities. This self-reported physical activity measure has previously shown predictive validity for mortality and other clinical risk factors in the Whitehall II cohort study (21, 22).

The total number of hours per week in moderate-to-vigorous physical activity (≥3 MET) was also calculated. Participants were considered to be meeting recommendations for moderate-to-vigorous physical activity if they reported engaging in ≥2.5 h of moderate or vigorous activity per week based on 2010 World Health Organization recommendations (23).

### Accelerometer-assessed physical activity

Participants with no contraindications (i.e., allergies to plastic or metal; traveling abroad over the following week) were asked to wear a wrist-worn triaxial accelerometer (GENEActiv; Activinsights Ltd.) on their nondominant wrist, nonstop, for 9 consecutive (24 h) days. The accelerometer was sampled at 87.5 Hz and data were stored in gravity (g) units (1 g = 9.81 m/s^2^). Calibration error was estimated based on static periods in the data and corrected if necessary (24). The Euclidean norm (magnitude) of the 3 raw signals minus 1 g, with negative numbers rounded to zero, was used to quantify acceleration related to the movement registered and expressed in milligravity units (12, 25).

Accelerometer data were processed in R by using the GGIR package (http://cran.r-project.org). Data extracted between the first and last midnight were retained for analyses, leading to a maximum of 24-h measurements for 8 d. Participants with valid data (≥16 h/d) for ≥2 weekdays and 2 weekend days were included in the analyses. Nonwear time was estimated on the basis of the SD and value range of each accelerometer axis, calculated for moving windows of 60 min with 15-min increments (16). For each 15-min period of time detected as nonwear time over the valid days, missing data were replaced by the mean acceleration calculated from measurement on other days at the same time of day (12, 24); a person-specific method, which is less prone to bias than methods that assume nonwear time reflects inactivity or is representative of the rest of the day (26). For each participant, duration in moderate-to-vigorous physical activity was also calculated. No established cutoff for moderate-to-vigorous physical activity using wrist acceleration in older adults is currently available; thus, we chose a 100-milligravity threshold based on the fact that walking at 4 km/h is classified as moderate physical activity (20) and was equivalent to an acceleration of 100 milligravity units in a laboratory-based study on 30 adults (27). To qualify as moderate-to-vigorous activity, ≥80% of the activity needed to be ≥100 milligravity units, for at least a period (bout) of 10 min by using moving 10-min windows.

Because the observation period covered 8 d, the data were recoded so that our measure reflected physical activity over 1 wk to match questionnaire-assessed physical activity. If a participant had 3 valid weekend days or 6 weekdays, the wrist acceleration of the first and last full day of measurement (e.g., 2 Tuesdays a week apart) were averaged to represent one unique day. Thus, the mean accelerometer-assessed total physical activity (milligravity) over 1 wk was calculated as follows: [(5 × mean daily weekday wrist acceleration) + (2 × mean daily weekend wrist acceleration)]/7. The same rescaling was done for duration in moderate-to-vigorous physical activity per week (min/wk).​ Participants undertaking ≥2.5 h of moderate or vigorous activity per week were considered to meet 2010 World Health Organization recommendations (23).

### Metabolic phenotypes of obesity

Objective anthropometric and metabolic risk factor data were assessed by a nurse in 2012–2013. BMI was calculated by using the following standard formula: weight (kg)/height (m)^2^. Participants were grouped as normal weight (BMI 18.5 to <25), overweight (BMI 25 to <30), or obese (BMI ≥30) based on World Health Organization International Classifications (28). Underweight participants (BMI <18.5) were excluded from the analyses. Participants were considered healthy (9) if they had <2 of the following 5 risk factors: HDL cholesterol <1.03 mmol/L for men and <1.29 mmol/L for women or taking lipid-lowering medication; blood pressure ≥130/85 mm Hg or taking antihypertension medication; fasting plasma glucose ≥5.6 mmol/L or taking diabetic medication; triacylglycerol ≥1.7 mmol/L; HOMA-IR >5.12 (90th percentile value in 2012–2013).

### Covariates

Covariates were assessed in 2012–2013. Basic demographic data included age, sex, ethnicity (white, nonwhite), and socioeconomic status as indicated by British civil service occupational position (administrative, professional/executive, clerical/support). Because many participants entered retirement before the 2012–2013 assessment (*n* = 2246, or 65% of the sample), data on preretirement occupational position was used from the 2002–2004 assessment if required. Health behaviors included cigarette smoking status (never smoker, ex-smoker, current smoker), alcohol consumption in the previous week (abstainer, 0 units/wk; moderate, 1–14 units/wk for women and 1–21 units/wk for men; high, >14 units/wk for women and >21 units/wk for men), and an indicator of diet quality based on 3 components used previously in the Whitehall II cohort (29). Participants were assigned an individual diet component score of 0 for each of the following: consuming fruit and vegetables less than daily, consuming whole/full-cream milk most often, and consuming white bread most often; a score of 1 for each of the following: consuming fruit and vegetables daily, consuming semiskim milk most often, and consuming a combination of white and brown bread or not consuming bread; and a score of 2 for each of the following: consuming fruit and vegetables twice or more per day, consuming skim/fat-free milk or other kind of milk most often, and consuming whole-meal, granary, or other brown bread most often. A total diet score was obtained by summing these individual diet components (range: 0–6). Participants were considered to have an unhealthy diet if this total diet score was between 0 and 2, a moderately healthy diet if this was between 3 and 4, and a healthy diet if this was between 5 and 6. If health behavior data were missing at the 2012–2013 assessment (*n* = 84, or 2% of the sample), data from the previous assessment (2007–2009) were used. Health status was indicated by responses to 2 questions on the presence of an illness that limits moderate or vigorous physical activity (grouped into does not limit at all, limits a little, limits a lot). Sleep duration was assessed by asking participants how many hours they sleep on an average weeknight (≤5 h, 6 h, 7 h, 8 h, ≥9 h).

### Statistical analyses

Questionnaire- and accelerometer-assessed total physical activity variables were standardized by using *z* scores (mean ± SD: 0.00 ± 1.00) to allow comparison between measures. Regression coefficients from general linear models and accompanying 95% CIs were used to examine cross-sectional differences in questionnaire- and accelerometer-assessed total physical activity across 6 phenotypes: healthy normal weight (reference group), unhealthy normal weight, healthy overweight, unhealthy overweight, healthy obese, and unhealthy obese. The first model adjusted for demographic factors. The second model further adjusted for socioeconomic position, health behaviors, and presence of an illness that limits moderate or vigorous activity.

Because the prevalence of metabolic risk factors was high (>20% for most factors), ORs are likely to inflate approximations of relative risk (30). Thus, Poisson models with robust error variances were used to examine prevalence ratios (PRs) for obesity and for each individual metabolic risk factor (hypertension, hyperglycemia, hyperlipidemia, insulin resistance, low HDL cholesterol) associated with an SD increase in accelerometer- and questionnaire-assessed total physical activity.

Similarly, because the number of participants undertaking ≥2.5 h/wk of moderate-to-vigorous physical activity was also high (54.5% based on questionnaire; 28.9% based on accelerometer), Poisson models with robust error variances were also used to estimate PRs for meeting recommendations for each group compared with healthy normal-weight adults. Akaike Information Criteria statistics were used to compare the fit of models based on questionnaire and accelerometer assessments.

In sensitivity analyses, we compared the likelihood of meeting physical activity recommendations between metabolic groups using data on moderate-to-vigorous physical activity in bouts of ≥1 min (instead of ≥10 min in the main analyses). We also repeated each analysis using a more stringent cutoff of 120 mg (instead of 100 mg) to define moderate-to-vigorous physical activity. The analyses were performed by using Stata 13 (StataCorp), and a 2-tailed *P* value <0.05 indicated statistical significance.

## RESULTS

### Sample characteristics

Of the 4880 participants to whom the accelerometer was proposed, 210 had contraindications, 4282 consented, and 4040 had valid accelerometer data (≥16 h/d) for ≥2 weekdays and 2 weekend days. Of those, 3457 participants also had data on questionnaire-assessed physical activity, BMI, metabolic risk factors, and covariates. Of the 3457 participants included in the analyses, 3359 (97.2%) participants had data for >16 h/d for the full 8 d, 43 (1.2%) for 7 d, 20 (0.6%) for 6 d, and 34 (1.0%) for 4 to 5 d. In all, missing data were replaced on average for 0.3% of the observation period, and 103 (3%) participants had missing data replaced for 1% to 5% of the period and 22 (0.6%) for 5% to 27% of the period.

Compared with participants included in the analytic sample, those excluded (*n* = 1423) were older (mean ± SD: 69.6 ± 5.9 compared with 69.2 ± 5.6 y; *P* = 0.008), more likely to be female (36.6% compared with 23.3%; *P* < 0.001), more likely to be of nonwhite ethnicity (10.8% compared with 6.8%; *P* < 0.001), and more likely to be of the lowest occupational position (11.8% compared with 7.8%; *P* < 0.001). Descriptive characteristics of the analytic sample are shown in **Table 1**.

**TABLE 1:** Characteristics of the sample of adults aged 60–82 y (77% male) in the Whitehall II cohort study by metabolic and obesity status (*n* = 3457)^1^

	Healthy normal weight(*n* = 864)	Unhealthy normal weight (*n* = 466)	Healthy overweight(*n* = 650)	Unhealthy overweight (*n* = 861)	Healthy obese (*n* = 161)	Unhealthy obese(*n* = 455)
Female, *n* (%)	238 (27.5)	87 (18.7)*	137 (21.1)*	137 (15.9)*	78 (48.4)*	130 (28.6)
Age, y	68.6 ± 5.7^2^	70.6 ± 5.6*	68.5 ± 5.4	70 ± 5.6*	68.2 ± 5.0	68.6 ± 5.5
Nonwhite ethnicity, *n* (%)	36 (4.2)	49 (10.5)*	23 (3.5)	75 (8.7)*	9 (5.6)	42 (9.2)*
Lowest occupational class, *n* (%)	44 (5.1)	38 (8.2)*	52 (8.0)*	56 (6.5)	22 (13.7)*	57 (12.5)*
Unhealthy diet, *n* (%)	81 (9.4)	63 (13.5)*	96 (14.8)*	130 (15.1)*	20 (12.4)	70 (15.4)*
Consumes fruit and vegetables < daily, *n* (%)	132 (15.3)	88 (18.9)	134 (20.6)*	196 (22.8)*	38 (23.6)*	110 (24.2)*
Consumes whole/full-fat milk most often, *n* (%)	95 (11.0)	33 (7.1)*	54 (8.3)	60 (7.0)*	7 (4.3)*	41 (9.0)
Consumes white bread most often, *n* (%)	108 (12.5)	82 (17.6)*	118 (18.2)*	158 (18.4)*	26 (16.1)	80 (17.6)*
Current smoker, *n* (%)	18 (2.1)	12 (2.6)	21 (3.2)	32 (3.7)*	5 (3.1)	15 (3.3)
High alcohol consumption in previous week, *n* (%)	101 (11.7)	61 (13.1)	81 (12.5)	135 (15.7)*	24 (14.9)	86 (18.9)*
Sleeps ≤5 h/night, *n* (%)	40 (4.6)	26 (5.6)	45 (6.9)	80 (9.3)*	11 (6.8)	48 (10.6)*
Has illness that greatly limits moderate or vigorous activity, *n* (%)	236 (27.3)	150 (32.2)	195 (30.0)	328 (38.1)*	80 (49.7)*	245 (53.8)*
Systolic blood pressure, mm Hg	121.2 ± 14.6	130.9 ± 16.2*	124.2 ± 13.7*	129.5 ± 16.6*	125.7 ± 14.3*	130.4 ± 15.4*
Diastolic blood pressure, mm Hg	67.9 ± 9.1	70.7 ± 10.3*	70.7 ± 9.2*	71.1 ± 10.1*	73.2 ± 9.1*	72.5 ± 9.7*
Fasting glucose, mmol/L	5.0 ± 0.4	5.7 ± 1.5*	5.1 ± 0.4	5.8 ± 1.6*	5.1 ± 0.4	6.1 ± 2.0*
HOMA-IR	1.2 ± 0.7	2.7 ± 3.2*	1.9 ± 1.0*	3.9 ± 6.5*	2.5 ± 1.3*	6.4 ± 10.2*
Triacylglycerol, mmol/L	0.9 ± 0.4	1.1 ± 0.6*	1.1 ± 0.4*	1.4 ± 0.8*	1.1 ± 0.3*	1.6 ± 0.9*
HDL cholesterol, mmol/L	1.9 ± 0.5	1.7 ± 0.5*	1.6 ± 0.4*	1.5 ± 0.4*	1.7 ± 0.4*	1.4 ± 0.4*
BMI, kg/m^2^	22.6 ± 1.6(18.52–24.99)^3^	23.3 ± 1.4(18.57–24.99)	27.1 ± 1.4(25.00–29.98)	27.3 ± 1.4(25.01–29.98)	32.6 ± 2.6(30.00–42.53)	33.6 ± 3.3(30.01–49.43)
Questionnaire-assessed physical activity						
Total physical activity,^4^ MET-h/wk	50.8 ± 28.5	47.7 ± 26.6*	48.2 ± 26.7	44.4 ± 25.8*	43.8 ± 23.3*	42.6 ± 25.9*
≥2.5 h/wk of moderate-to-vigorous activity,^5^ *n* (%)	509 (58.9)	270 (57.9)	385 (59.2)	447 (51.9)*	73 (45.3)*	201 (44.2)*
Accelerometer-assessed physical activity						
Total physical activity (weekly acceleration), milligravity	25.8 ± 7.4	23.3 ± 6.5*	24 ± 6.1*	22.5 ± 5.9*	22.3 ± 6.0*	20.6 ± 5.3*
≥2.5 h/wk of moderate-to-vigorous activity,^5^ *n *(%)	368 (42.6)	128 (27.5)*	196 (30.2)*	201 (23.3)*	32 (19.9)*	74 (16.3)*

1 *Significantly different from healthy normal weight, *P* < 0.05 (linear or logistic regression). MET, metabolic equivalent.

2 Mean ± SD (all such values).

3 Mean ± SD; range in parentheses (all such values).

4 Defined as sum of [activity duration (h/wk) × MET score].

5 2010 World Health Organization guidelines (23).

### Associations between total physical activity and healthy obesity

Spearman’s correlation between self-reported and objectively assessed total physical activity appeared to be nondifferential between healthy normal-weight (0.300, *P* < 0.001) and healthy obese (0.296, *P* < 0.001) adults. This correlation was weaker within unhealthy obese adults (0.205, *P* < 0.001).

**FIGURE 1:**
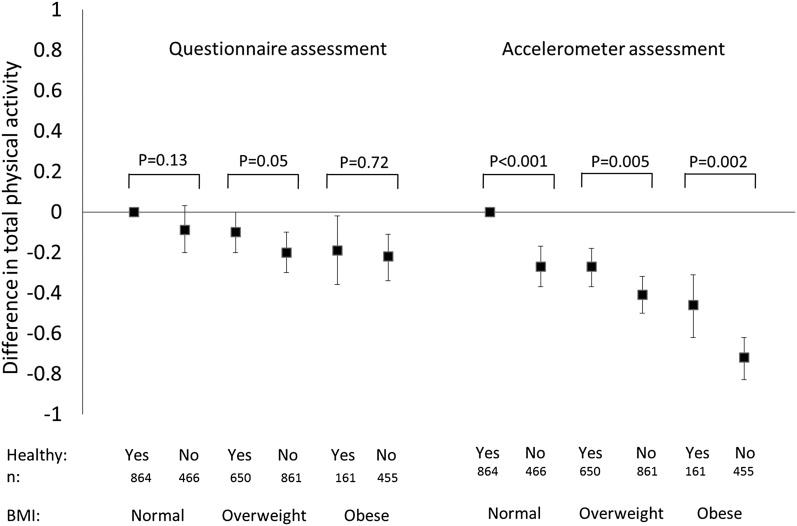
Differences in total physical activity across metabolic and obesity phenotypes based on questionnaire and accelerometer assessments in the Whitehall II cohort study (*n* = 3457). Data are standardized *z* scores to allow comparability between measures. Models were adjusted for age, sex, ethnicity, occupational position, diet quality, smoking status, alcohol consumption, sleep duration, and presence of an illness that limits moderate or vigorous activity. Model fit was better with the accelerometer-based than with the questionnaire-based assessments (Akaike Information Criterion for fully adjusted models = 9149.87 and 9707.06, respectively).

Based on the questionnaire measure, total physical activity was lower in unhealthy overweight, healthy obese, and unhealthy obese adults than in healthy normal-weight adults in models adjusted for age, sex, and ethnicity (**Supplemental Table 1**). In models further adjusted for occupational position, health behaviors, and health status (**Figure 1**; Supplemental Table 1), unhealthy overweight, healthy obese, and unhealthy obese adults had −0.20 (−0.30, −0.10), −0.19 (−0.36, −0.02), and −0.22 (−0.34, −0.11) lower SD units of questionnaire-assessed total physical activity compared with healthy normal-weight adults, respectively. Pairwise comparisons showed no differences in questionnaire-assessed total physical activity between metabolically healthy and unhealthy adults within any BMI category in the fully adjusted models.

All groups had lower accelerometer-assessed total physical activity than healthy normal-weight groups, both in minimally adjusted models and after further adjustments for occupational position, health behaviors, and health status (Figure 1; Supplemental Table 1); total physical activity was −0.46 (−0.62, −0.31) SD units lower in healthy obese adults and −0.72 (−0.83, −0.62) SD units lower in unhealthy obese adults. Pairwise comparisons showed differences in total physical activity between healthy and unhealthy groups within all BMI categories at both stages of adjustment; healthy obese adults had higher total physical activity than their unhealthy obese counterparts (*P* = 0.002). Differences in total physical activity between healthy and unhealthy adults within each BMI group remained significant after multiple comparisons were accounted for (0.05 divided by 5, for 5 comparisons, yields a significance threshold of *P* < 0.01). Further adjustment for BMI did not eliminate the significance of differences within any group (*P* < 0.001 between normal-weight groups; *P* = 0.01 between overweight groups; *P* = 0.01 between obese groups). The overall fit of the model was better with accelerometer- than with questionnaire-based assessments (Figure 1).

**FIGURE 2:**
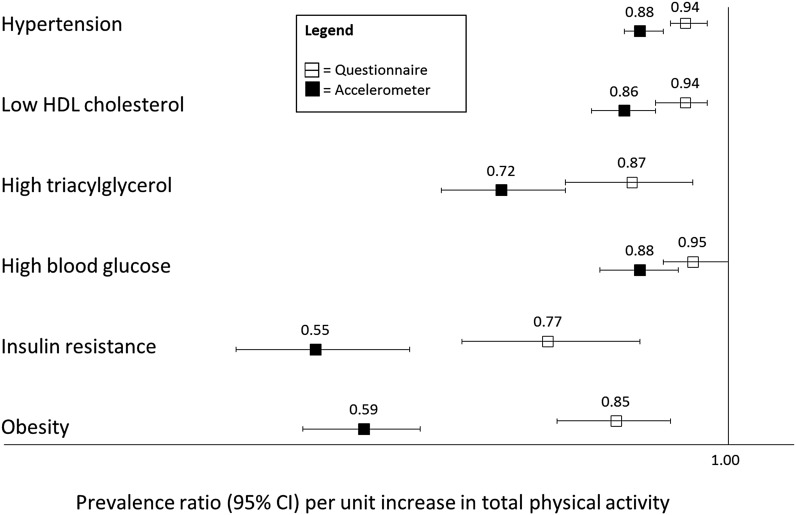
Association of questionnaire- and accelerometer-assessed total physical activity and individual metabolic risk factors in the Whitehall II cohort study (*n* = 3457). Data are standardized *z* scores to allow comparability between measures. Case numbers (*n*) are as follows: hypertension = 2161; low HDL = 1593; high triacylglycerol = 572; high blood glucose = 1071; insulin resistance = 344; obesity = 616. Prevalence ratios are based on Poisson regression models with robust error variances. Models were adjusted for age, sex, and ethnicity. Reference groups reflect the absence of each individual metabolic risk factor under consideration.

Associations of increasing total physical activity with reduced prevalence of each individual metabolic risk factor were consistently stronger with accelerometer- than with questionnaire-based assessments; insulin resistance showed the greatest reduction in prevalence with higher accelerometer-assessed total physical activity (**Figure 2****; Supplemental Table 2**).

### Associations between moderate-to-vigorous physical activity and healthy obesity

In comparison with healthy normal-weight adults, the prevalence of meeting recommendations for moderate-to-vigorous physical activity as assessed by questionnaire was lower among unhealthy overweight, healthy obese, and unhealthy obese adults when adjusted for age, sex, and ethnicity (**Table 2**). After further adjustment for other covariates, the prevalence among unhealthy overweight (PR: 0.91; 95% CI: 0.84, 0.99) and unhealthy obese (PR: 0.85; 95% CI: 0.76, 0.96) adults decreased, whereas healthy obese adults were not less likely to meet recommendations than were healthy normal-weight adults. Models with unhealthy obese adults as the reference group (**Supplemental Table 3**) indicated that healthy obese adults were not more likely to report ≥2.5 h of moderate-to-vigorous physical activity per week than were unhealthy obese adults.

**TABLE 2:** Likelihood of meeting 2010 World Health Organization recommendations for moderate-to-vigorous physical activity compared with healthy normal-weight adults, based on questionnaire and accelerometer assessments in the Whitehall II cohort study (*n* = 3457)^1^

	Meets physical activity recommendations based on questionnaire		Meets physical activity recommendations based on accelerometer	
	Model 1^2^	Model 2^3^	Model 1^2^	Model 2^3^
Metabolic and obesity status				
Healthy normal weight (*n* = 864)	1.00 (reference)	1.00 (reference)	1.00 (reference)	1.00 (reference)
Unhealthy normal weight (*n* = 466)	1.00 (0.91, 1.10)	1.01 (0.92, 1.11)	0.74 (0.63, 0.87)	0.75 (0.64, 0.89)
Healthy overweight (*n* = 650)	0.98 (0.90, 1.07)	1.01 (0.93, 1.10)	0.69 (0.60, 0.79)	0.71 (0.62, 0.81)
Unhealthy overweight (*n* = 861)	0.88 (0.81, 0.95)	0.91 (0.84, 0.99)	0.59 (0.51, 0.68)	0.63 (0.55, 0.72)
Healthy obese (*n* = 161)	0.82 (0.69, 0.98)	0.89 (0.75, 1.06)	0.50 (0.37, 0.68)	0.59 (0.43, 0.79)
Unhealthy obese (*n* = 455)	0.77 (0.68, 0.86)	0.85 (0.76, 0.96)	0.39 (0.32, 0.49)	0.46 (0.37, 0.58)
Akaike Information Criterion	5992.47	5963.40	4226.51	4183.95

1 Physical activity recommendations are based on 2010 World Health Organization guideline (23) of ≥2.5 h/wk of moderate-to-vigorous physical activity in bouts of ≥10 min. Values are prevalence ratios (95% CIs) based on Poisson regression models with robust error variances.

2 Adjusted for age, sex, and ethnicity.

3 Adjusted as for model 1 and for occupational position, diet quality, smoking status, alcohol consumption, sleep duration, and presence of an illness that limits moderate or vigorous activity.

The prevalence of undertaking ≥2.5 h/wk of moderate-to-vigorous activity when assessed by accelerometer was lower in all phenotypes than in healthy normal-weight adults at both stages of adjustment (Table 2). In comparison with healthy normal-weight adults, the prevalence was 0.59 (0.43, 0.79) times lower in healthy obese adults and 0.46 (0.37, 0.58) times lower in unhealthy obese adults after adjustment for all covariates. Models with the unhealthy obese as the reference group (Supplemental Table 3) indicated that healthy obese adults were not more likely to perform ≥2.5 h/wk of accelerometer-assessed moderate-to-vigorous physical activity than were unhealthy obese adults. The overall fit of the model was better when accelerometer rather than questionnaire assessments were used.

Sensitivity analyses using a more stringent 120-mg cutoff for accelerometer-assessed moderate-to-vigorous physical activity produced results of a pattern and magnitude similar to that of the main analyses (**Supplemental Table 4**). Healthy obese adults were not more likely to demonstrate ≥2.5 h/wk of moderate-to-vigorous activity than unhealthy obese adults (PR: 1.22, 95% CI: 0.77, 1.92) when this activity was restricted to bouts of ≥10 min. However, healthy obese adults were more likely than unhealthy obese adults to meet recommendations when moderate-to-vigorous activity as measured with the more stringent 120 mg cutoff was expanded to include ≥1-min bouts (PR: 1.31; 95% CI: 1.05, 1.64; **Supplemental Table 5**).

## DISCUSSION

Total physical activity was higher among metabolically healthy obese adults than among their unhealthy obese counterparts, and these differences were evident only when measured objectively. Increased total physical activity was associated with a reduced prevalence of all metabolic risk factors individually, and the largest reduction was observed for insulin resistance. Healthy obese adults were not more likely than unhealthy obese adults to meet current recommendations for moderate-to-vigorous physical activity (requiring 10-min bouts).

Associations of total physical activity with phenotypes were consistently stronger when accelerometer- rather than questionnaire-based assessments were used, which is likely explained by the fact that physical activity is often of an incidental nature and difficult to recall. Weak or null findings on total physical activity in previous studies may have been due to reliance on imprecise questionnaire-based measures of physical activity. A hip-worn counts-based accelerometer was previously used to show that total physical activity was lower in healthy overweight and obese adults than in healthy normal-weight adults (31), but no comparisons were made with unhealthy groups. One other study using a pedometer (step count) did not find differences in total physical activity between healthy and unhealthy obese groups, but this was based on a small sample of obese women (*n* = 39) (7). Overall, there was a low-to-moderate correlation between self-reported and objectively assessed total physical activity, with a lower correlation found among unhealthy than among healthy obese adults, which suggests differential measurement error between obese groups. These results might explain the weaker associations observed with self-reported data.

Our interpretations are not straightforward, however, because our wrist-worn accelerometer captured total movement over full 24-h periods and thus considered all of sedentary behavior, sleep duration, light, moderate, and vigorous intensity activities. Light-intensity physical activity may be associated with metabolic risk factor clustering independently of moderate-to-vigorous intensity activities (14). Sedentary behavior is also independently associated with metabolic risk factors (32), but has little support as a separate determinant of healthy obesity. No differences in sitting time were observed between healthy and unhealthy obese adults, as indicated by self-reported television viewing or other sedentary activities in 2 studies (10, 33); however, one study reported lower sedentariness among healthy than among unhealthy obese adults based on crude analyses of self-reported activity duration, intensity, and frequency (11). In addition, unhealthy obese women reported a shorter daily sleeping duration than did their healthy obese counterparts in one study (10). Short sleeping duration may exert adverse effects on glucose tolerance, insulin resistance, and risk of type 2 diabetes either directly or indirectly through disrupting appetite regulation (34). A self-reported measure of sleep duration was used in the current study; shorter sleep duration was more common in unhealthy than in healthy obese adults. However, sleep duration did not explain differences in total physical activity between obese groups.

There was no clear difference in the likelihood of meeting recommendations for moderate-to-vigorous physical activity between healthy and unhealthy obese adults. A difference was observed only when durations of moderate-to-vigorous physical activity as measured with a more stringent cutoff (which is more likely to capture activities in the more intense end of the moderate-to-vigorous distribution and to be less contaminated by light intensity activity) and with durations of ≥1 min instead of 10 min were considered. This suggests that healthy obese adults do engage in more moderate-to-vigorous activity, but in shorter durations, which are unrecognized by current guidelines. Additional research is needed to confirm this finding.

Confounding effects of diet are difficult to control for in observational studies. There are known protective effects of fruit, vegetable, and whole-grain consumption against abnormal glucose metabolism and risk of type 2 diabetes (35, 36) and apparently inverse associations of high-fat diary intake with obesity and metabolic ill health (37). Differences in total physical activity between all healthy and unhealthy BMI groups were evident after control for these indicators of diet quality, which supports an independent effect of physical activity on metabolic risk factors. These findings also support the notion that higher physical activity, even in short durations, along with diet will improve metabolic health among obese adults.

The need for a standardized definition of “metabolically healthy” obesity has been emphasized repeatedly, most recently in the World Obesity Forum’s 2013 Stock Report (38). Our definition of a “healthy” metabolic profile was based on criteria used previously in the US NHANES study (9), which considers core metabolic risk factors of hypertension, blood lipids, and blood glucose, but additionally includes insulin resistance, which is excluded in widely used Adult Treatment Panel III criteria (39). Waist circumference was excluded from our criteria because it is highly correlated with BMI (40). However, a range of metabolic risk factors, including inflammation and inefficient mitochondrial transcription, are closely aligned with the presence of liver fat in obesity (41), and liver fat may increase the risk of incident type 2 diabetes in healthy obese adults (42). Ectopic fat may therefore be a paramount target for disease risk reduction in obese and nonobese groups. Importantly, physical activity has the potential to improve fat distribution by reducing visceral fat, even in the absence of weight loss (43). A more favorable fat distribution has been associated with increased maintenance of a metabolically healthy profile in obese adults over a period of 10 y and elimination of excess risk of type 2 diabetes and cardiovascular disease (11).

### Strengths and limitations

The strengths of this study include the objectively measured anthropometric and metabolic risk factors and the use of a novel triaxial accelerometer device worn on the wrist to objectively measure physical activity in adults of different obesity phenotypes, which afforded greater precision in effect estimates. We had the advantage of making direct comparisons between self-reported questionnaire- and objective wrist-worn accelerometer-based assessments of total and moderate-to-vigorous physical activity, providing insight into their relative strength and utility. Health behavior and health status covariates were based on self-reported data, however, and are thus subject to measurement inaccuracies. This study was cross-sectional and thus was not able to determine whether increased physical activity preceded or was preceded by metabolic phenotypes of obesity; however, adjustment for the presence of an illness that limits moderate-to-vigorous activity partly controlled for reverse causation. Accelerometry results are subject to the specific brand and placement used in the current study; however, strong correlations have been found with oxygen uptake for both GeneActiv and Actigraph accelerometers and for both wrist and hip placements (27). Participants were of an older age, and results cannot be readily generalized to younger or middle-aged adults.

### Conclusions

Healthy obese adults have higher total physical activity than do unhealthy obese adults, and these differences are evident only when measured objectively with a wrist-worn accelerometer. Physical activity likely has a greater role in promoting health in obese populations than previously thought and may confer substantial reductions in disease burden. Future research could examine prospectively whether increases in physical activity in unhealthy obese adults lead to a healthier status.
